# La gigantomastie gravidique à l'Institut du Cancer de Dakar: à propos de 2 cas

**DOI:** 10.11604/pamj.2015.22.314.8085

**Published:** 2015-11-27

**Authors:** Sidy Ka, Jaafar Thiam, Maimouna Mane, Rolland Some, Rokhaya Niang, Céline Goudiaby, Ahmadou Dem

**Affiliations:** 1 Institut Joliot Curie, Dakar, Sénégal

**Keywords:** Gigantomastie, grossesse, traitement hormonal, réduction mammaire, gigantomastia, pregnancy, hormonal treatmentl, breast reduction

## Abstract

La gigantomastie gravidique est une augmentation exagérée et invalidante de la taille des seins survenant pendant la grossesse chez une patiente aux seins préalablement normaux. Sa physiopathologie est mal cernée. Elle pose localement des problèmes trophiques et rend difficile la grossesse. Le traitement est médical anti hormonal et chirurgical sur la base d'une réduction mammaire. Il est difficile et peut compromettre l'avenir esthétique et fonctionnel de la glande mammaire. Nous rapportons 2 cas de gigantomasties gravidiques suivies et traitées à l'Institut Joliot Curie de Dakar.

## Introduction

La gigantomastie gravidique se définit comme une augmentation invalidante de la taille des 2 seins survenant pendant la période de la grossesse [1]. Il est du à des phénomènes hormonaux sur des terrains mal définis de mastopathies [2]. Le tableau clinique est bruyant et la patiente voit ses seins grossir rapidement pour devenir inflammatoires et douloureux avec un poids difficile à supporter rendant les gestes de tous les jours et la grossesse pénibles [3]. Peu de cas ont été rapportés concernant la femme noire. Nous rapportons 2 cas de gigantomasties gravidiques suivies à l'Institut Joliot Curie de Dakar.

## Patient et observation

### Observation 1

**Figure 1:**
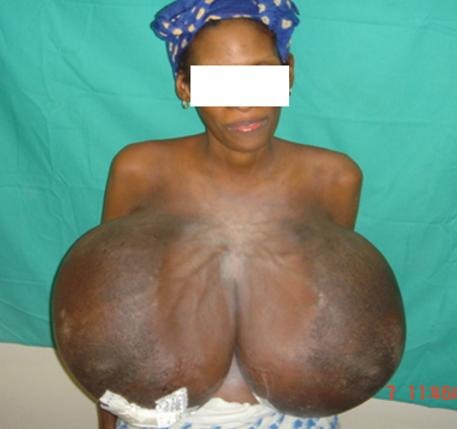
Volumineuse hypertrophie mammaire bilatérale et éléphantiasiforme

**Figure 2:**
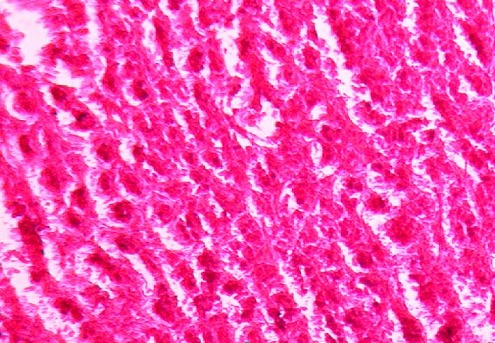
Grossissement X 100. Hyperplasie des cellules des unités ducto-lobulaires dans un stroma palléal fibreux par endroits

Il s'agissait d'une patiente de 33 ans, troisième geste primipare avec des ménarches à 16 ans qui a été reçue à 15 semaines d'aménorrhée pour une augmentation rapide, douloureuse et bilatérale des seins. L'examen montrait des seins volumineux arrondis de la taille de ballons de basket avec une peau luisante piquetée en peau d'orange et des aires ganglionnaires libres (Figure 1). L'examen obstétrical était normal. La cytologie évoquait un carcinome tandis que l'histologie sur la base d'une biopsie mammaire montrait une mastosefibro hyperplasique bilatérale (Figure 2). L'imagerie ne suspectait pas de cancer. Le bilan biologique montrait une hyperprolactinémie et un taux de FSH et LH à 6,77 et 3,45 UI/ml. Une TDM ne montrait pas d'anomalies au niveau de la selle turcique. Elle a bénéficié d'un traitement à base de bromocriptine qui n'améliorait pas la symptomatologie. La grossesse s'est arrêtée spontanément. Une mastectomie bilatérale a été effectuée. Les suites opératoires étaient simples. Elle a contracté une grossesse menée à terme après 14 mois de suivie.

### Observation 2

**Figure 3:**
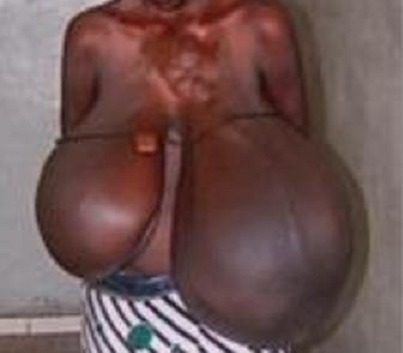
Volumineuse hypertrophie mammaire bilatérale et éléphantiasiforme

**Figure 4:**
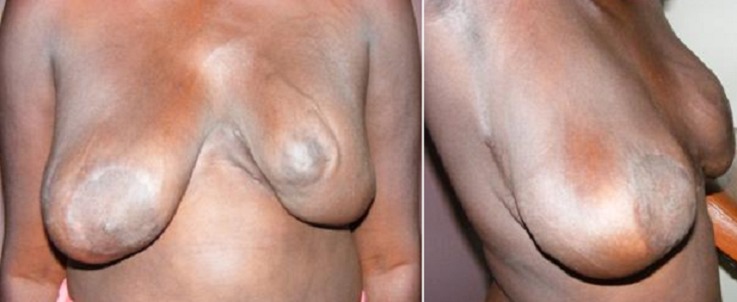
Réduction mammaire pour gigantomastie gravidique. Vues de face et de profil à 12 mois

Il s'agissait d'une patiente de 21 ans primi-geste reçue à 20 semaines d'aménorrhées pour une augmentation brutale, douloureuse et bilatérale des seins, modifiant la marche entraînant une fatigabilité importante. L'examen montrait des seins de volume important mesurant du sillon mammaire ou mamelon 52 cm à gauche et 43 cm à droite. Les seins étaient parcourus de veines sous cutanées dilatées avec épaississement éléphantiasiforme de la plaque aréolo-mammelonnaire (Figure 3). La pression du sein montrait du colostrum d'aspect normal. On retrouvait au niveau axillaire des ganglions augmentés de volume d'aspect réactionnel. Une échographie mammaire montrait un épaississement cutané et une glandemammaire siège de lésions nodulaires bien limitées et œdémateuses. Une biopsie montrait une hyperplasie épithéliale bénigne. Le taux de prolactine était très élevé pour le terme. L'examen des autres appareils était normal. L'examen obstétrical et l'imagerie fœtale et des annexes montraient une grossesse évolutive de 20 semaines d'aménorrhées avec un pronostic favorable. Nous avons opté pour conduire la grossesse à 32 semaines et procéder à une extraction ce qui a été accomplie. L'enfant se portait bien avec un poids de 2700 grammes et un score d'Apgar à 9. Nous avons effectué un traitement avec de la bromocriptine qui a très peu amélioré le tableau. Elle a bénéficié d'une chirurgie de réduction mammaire. A moyen terme elle a présenté un lâchage partiel de sutures et à long terme une réduction du volume mammaire et une cicatrisation satisfaisante (Figure 4).

## Discussion

La gigantomastie gravidique est une affection rare. Au Sénégal 3 cas ont été documentés [2]. Son incidence est estimée à un cas pour 30 000 à 100 000 grossesses [1]. Elle serait liée à une exagération des phénomènes hyperplasiques physiologiques de la grossesse conduite par une augmentation des récepteurs aux œstrogènes ou à la progestérone. Ceci n'est pas décrit par tous les auteurs [1, 4]. L'Age de survenue n'est pas spécifique. Elle est cependant fréquente chez la multipare après des premières grossesses normales [5]. Nos patientes étaient jeunes et ne présentaient pas d'antécédents particuliers. L'une était multipare tandis que l'autre était primipare. Le diagnostic se fait sur un faisceau d'argument. Dans tous les cas il est marqué par une augmentation très rapide du volume mammaire supérieur à 1500 cc [2]. Elle est essentiellement bilatérale avec la présence d'un aspect inflammatoire avec épaississement cutané et la survenue d'une turgescence des veines du sein. L'imagerie est pauvre. L’échographie est l'examen de routine le plus performant en situation gravidique et inflammatoire. Elle montre un épaississement œdémateux des structures cutanées et glandulaires [1, 2]. Elle permet de caractériser ou de dépister des lésions associées [6]. Le bilan biologique montre une augmentation variable de la prolactinémie non liée à une anomalie hypophysaire. Elle était importante chez une de nos patientes. L'histologie nécessaire devant une augmentation inflammatoire des seins montre une prolifération épithéliale pluristratifiée floride avec des structures papillaires, sans atypie, les cellules épithéliales sont vacuolisées témoignant d'une activité sécrétoire importante. Au niveau du stroma on retrouve œdème, sclérose et nécrose [2, 7]. L'aspect caricatural dans ce contexte ne doit pas occulter la possibilité d'autres mastopathies rares comme les lymphomes mammaires [7]. C'est une affection mal tolérée à cause des douleurs qui sont fréquentes [3]. Dans un cas notre patiente avait des douleurs rachidiennes et des difficultés à marcher à cause d'une cyphose qui mettait le rachis en conflit avec la lordose physiologique de la grossesse. Le pronostic de la grossesse n'est cependant pas affecté [2].

Le traitement n'est pas bien codifié. Des traitements médicaux, obstétricaux et chirurgicaux ont été proposés [8, 9]. Il dépend des équipes, du terme,du pronostic de la grossesse, et des troubles trophiques mammaires. En début de grossesse certains auteurs ont proposé une interruption thérapeutique de la grossesse. Elle se heurte au choix de la patiente et de l’équipe de soins et à des problèmes éthiques [8]. Au-delà du premier trimestre on préconise la surveillance jusqu’à maturation de l’œuf pour extraction chirurgicale. Des soins locaux sont administrés et une correction des déficits humoraux chez la mère [2]. L'absence derégression significative sous bromocriptine fait proposer des options chirurgicales que sont la mastectomie et la réduction mammaire si l’état local le permet. La réduction utilise les techniques classiques telles que le « T » inversé et à pédicule supérieur ou inférieur. Mais sur les seins très volumineux, la tentative de conservation de la plaque aréolo-mamelonnaire est hasardeuse [10, 11]. La mastectomie peut être associée à une reconstruction mammaire autologue ou prothétique [12]. Chez une de nos patientes nous avons opté pour une mastectomie bilatérale. Chez la deuxième patiente nous avons tenté avec succès une réduction mammaire après césarienne. Le choix de la mastectomie est fait sur la possibilité de récidive et sur les difficultés liées à une intervention sur terrain de seins dystrophiques inflammatoires et engorgés. Tandis que le choix de la réduction mammaire est fait sur la possibilité d'une surveillance de la patiente et d'une option secondaire de mastectomie en cas d’échec ou de récidive [13–15]. Les traitements hormonaux à base d’œstrogène, de progestérone et de testostérone n'ont pas fait la preuve de leur efficacité [8]. Le pronostic est essentiellement local. On veillera à l'hygiène du sein et à un bon suivi obstétrical [2].

## Conclusion

La gigantomastie gravidique est une affection rare. Sa physiopathologie est mal élucidée. C'est une affection locale qui ne met pas en jeu le pronostic de la grossesse. Mais elle est très mal supportée et compromet l'avenir esthétique et fonctionnel des seins. Le diagnostic est aisé dans le contexte de grossesse, après élimination d'une tumeur inflammatoire bilatérale. Le traitement n'est pas bien codifié et dépend de l’état local et de considérations techniques physiologiques et éthiques propres à chaque équipe.

## References

[1] Boufettal H, Khalkane L, Dlia H, Mahdaoui S (2009). Gigantomastie gravidique bilatérale: à propos d'un cas. Jour Gyn Obs Biol Reprod..

[2] Dem A, Wone H, Faye ME, Dangou JM, Toure P (2009). la gigantomastie gravidique: à propos d'un cas. JournGynObstBiolReprod..

[3] Lapid O, de Groof EJ, Corion LU, Smeulders MJ, van der Horst CM (2013). The effect of breast hypertrophy on patient posture. Arch Plast Surg..

[4] Houssine B, Leila K, Mahdaoui S, Hermas S (2001). La gigantomastie gravidique bilatérale: à propos d'un cas. Imagerie de la femme..

[5] Chavoin JP, Canizares F, Mojallal A, Fabre G, Grolleau JL (2005). Hypotrophie et ptôse mammaire. Ann Chir Plast Esth..

[6] Schmid N, De Greef C, Calteux N, Duhem C, Faverly D (2008). Réduction mammaire selon la technique verticale pour gigantomastie avec fibroadénomatose massive: à propos d'un cas clinique. Ann Chir Plast Esth..

[7] Vandenberghe G, Claerhout F, Amant F (2005). Lymphoblastic lymphoma presenting as bilateral gigantomastia in pregnancy. Int J Gynaecol Obstet..

[8] Eben F, Cameron MD, Lowy C (1993). Successful treatment of mammary hyperplasia in pregnancy with bromocryptine. Br J ObstetGynaecol..

[9] Greely PW, Robertson LE, Curtin JW (1965). Mastoplasty for bilateral benign breast hypertrophy associated with pregnancy. Ann Surg..

[10] Segall JJ (2005). Plastie mammaire de réduction et mastopexie avec cicatrices verticale et horizontale limitées « mini T inversé »: expérience de 184 cas. EMC Chir..

[11] Ohana J, Karcenty B, Meouar R, Amar A (2013). L'utilisation des implants mammaires dans la ptôse, l'hypertrophie et les malformations du sein. Imagerie de la femme..

[12] Boyce SW, Hoffman PG, Mathes SJ (1984). Recurrent macromastia after subcutaneous mastectomy. Ann Plast Surg..

[13] Chargui R, Houimld S, Damak T, Khomsi F, Ben Hasouna J (2006). Récidive d'une gigantomastie après mammoplastie: à propos d'un cas et revue de la littérature. Ann Chir Plast Esth..

[14] Letertre P, Lasserre G, Ricbourg B (2009). Traitement des hypertrophies mammaires très importantes et des gigantomasties par la technique de réduction dite à pédicule postéro-inférieur: à propos de 20 cas. Ann Chir Plast Esth..

[15] Mojallal A, Comparin JP, Voulliaume D, Chichery A (2005). Place de la réduction mammaire à pédicule supérieur dans les gigantomasties. Ann Chir..

